# Valorization of Vetiver Root Biochar in Eco-Friendly Reinforced Concrete: Mechanical, Economic, and Environmental Performance

**DOI:** 10.3390/ma16062522

**Published:** 2023-03-22

**Authors:** Sameer Neve, Jiang Du, Rojyar Barhemat, Weina Meng, Yi Bao, Dibyendu Sarkar

**Affiliations:** Department of Civil, Environmental and Ocean Engineering, Stevens Institute of Technology, Hoboken, NJ 07030, USA

**Keywords:** biochar, circular economy, finite element analysis, green concrete, leaching of heavy metals, reinforced concrete

## Abstract

Biochar has shown great promise in producing low-cost low-carbon concrete for civil infrastructure applications. However, there is limited research comparing the use of pristine and contaminated biochar in concrete. This paper presents comprehensive laboratory experiments and three-dimensional nonlinear finite element analysis on the mechanical, economical, and environmental performance of reinforced concrete beams made using concrete blended with biochar generated from vetiver grass roots after the roots were used in an oil extraction process. Both pristine biochar and biochar that were used to treat wastewater through adsorbing heavy metals (100 mg/L of Pb, Cu, Cd, and Zn) were investigated. The biochar was used to replace up to 6% Portland cement in concrete. Laboratory experiments were conducted to characterize the workability, mechanical properties, shrinkage, and leaching potential of the concrete blended with biochar. The results showed that using biochar could increase the compressive strengths and reduce the shrinkage of concrete without causing a leaching problem. The results from finite element analysis of the reinforced concrete beams showed that the use of biochar was able to increase the flexural performance of the beams as well as their economic and environmental performance. This research will promote the development and structural applications of low-cost low-carbon concrete.

## 1. Introduction

Concrete is the most widely used construction material worldwide because of its low cost, ease of use, and local availability [1]. Concrete is produced with carbon-intensive ingredients such as Portland cement whose manufacturing process involves combustion at high temperatures and releases large amounts of CO_2_. The manufacturing of cement accounts for about 8% of global emissions caused by humans [2]. Substituting cement with low-carbon low-cost materials is promising to drastically lower the carbon footprint and cost of concrete for low-carbon cost-effective concrete structures.

Previous research showed that it is promising to valorize various types of waste to produce such materials to amend with concrete for structural applications. The widely used types of solid waste include industrial by-products (e.g., fly ash [3] and slag [4]), construction and demolition waste (e.g., waste concrete [5] and waste brick [6]), as well as municipal waste (e.g., waste glass [7] and waste plastic [8]). The various types of waste were utilized to replace Portland cement partially or fully. Extensive research has shown that the appropriate use of solid waste can improve the workability, mechanical properties, and durability of concrete while reducing the carbon footprint and material cost [9,10]. For example, Spiesz et al. used fly ash and slag into concrete blended with waste glass to mitigate the alkali-silica reaction, which is often induced by the waste glass, while sustaining the workability and mechanical properties of concrete [11]. Torres et al. discovered that using fine foundry waste improved the compressive, tensile, and flexural strengths of concrete [12]. Ling and Nor valorized waste tire to increase the skid resistance of concrete paving blocks [13]. Modarres et al. found that utilizing coal waste ash and coal waste powder in concrete pavement increased the compressive strength and toughness of concrete [14].

Recently, biochar made using lignocellulosic waste has attracted increasing interest because of its potential to improve concrete properties. First, using biochar can enhance the mechanical properties of concrete. For example, using biochar to partially replace Portland cement increased the compressive and flexural strengths of cement paste and mortar [15,16]. The enhancement of the mechanical strengths is attributed to the filler effect of fine biochar particles that filled pores and thus refined the microstructures [17]. The filler effect not only densifies the matrix but also enhances the interfacial transition zones when aggregates are utilized [18]. Second, biochar with macro-pores (diameter > 0.1 mm) imparts an internal curing effect. Pre-soaked biochar particles that absorbed water released into the matrix during the hardening process [18]. The released water mitigated the shrinkage of the matrix [19], which in turn helped minimize the occurrence of cracking [20]. Third, biochar is usually highly porous, as volatiles escape during pyrolysis, and, therefore, the use of biochar increases the thermal spalling resistance of concrete as the pores alleviate vapor pressure and the damages caused by the internal pressure at high temperatures [17]. In addition to the benefits of concrete properties, biochar gains dimensional strength and resistance to biological degradation through pyrolysis by hindering inflation because of the removal of cellulosic elements, particularly hemicellulose, which can absorb moisture and are vulnerable to biodegradation [17].

Previous studies by Tan et al. [21] indicate that the addition of low amounts of biochar (1–3% by mass) increased the compressive strength, and the addition of high amounts of biochar (>5% by mass) decreased the compressive strength. Whereas the increase in biochar dosage did not significantly impact the flexural strength. The addition of biochar to green roofs also worked efficiently as a water retention medium, leading to a reduction in roof temperature [22]. Biochar derived from date palm leaves and seeds as up to 1% cement replacement led to an increase in compressive strength by about 7% [23]. Recent research has shown that it is possible to achieve negative-carbon concrete with biochar which was used to sequestrate carbon dioxide [24,25].

The above studies on using biochar in concrete production demonstrate the feasibility of using biochar to improve the mechanical properties and durability of concrete. Since biochar is often considered waste, the use of biochar in producing concrete will likely reduce the carbon footprint and cost of concrete, simultaneously. Meanwhile, it is promising to use biochar to treat wastewater with heavy metals [26,27], because the high specific surface area of biochar promotes the adsorption and immobilization of heavy metals. It is rational to imagine whether the biochar used to adsorb heavy metals can be used to produce concrete for structural applications. Despite the potential benefits, several challenges have been identified from utilizing biochar to absorb heavy metals and produce concrete: (1) the presence of heavy metals may compromise the mechanical properties and durability of concrete since heavy metals often hinder the hydration reactions of cement [28]. (2) The constructional performance of concrete blended with biochar is still unclear because the porous microstructure of biochar may absorb the mixing water and affect the workability of concrete adversely. (3) The heavy metal leaching potential of concrete blended with biochar remains unclear, thus leading to concerns about the environmental performance of concrete for structural applications.

This research is motivated by the hypothesis that the combination of biochar and concrete can achieve desired constructional, mechanical, economic, and environmental performance through the appropriate design of the concrete mixture and reinforced concrete for structural applications. To test this hypothesis, this research has four main objectives: (1) to evaluate the efficiency of the vetiver root biochar in removing heavy metals in an aqueous media; (2) to evaluate the workability, compressive strengths, shrinkage, and leaching potential of concrete blended with biochar; (3) to understand the underlying mechanisms of the effect of biochar on concrete properties; and (4) to evaluate the mechanical, economic, and environmental performance of reinforced concrete made using the developed concrete with biochar.

These research objectives reflect the intellectual merits of this research, which generates new knowledge about the employment of biochar on the constructional, mechanical, economic, and environmental performance of reinforced concrete. Regarding the broader impacts, the knowledge generated from this research will promote the valorization of various types of waste in producing low-carbon cost-effective concrete for structural applications, which will, on one hand, reduce the carbon footprint and cost of the construction industry, and on the other hand, minimize the landfill of waste and the environmental impact such as leaching of heavy metals from waste.

To achieve the above research objectives, this research conducted comprehensive laboratory experiments on biochar and biochar-modified concrete to investigate the effects of both pristine and heavy metal-contaminated biochar on the fresh and hardened properties as well as the leaching potential of concrete and performed finite element analysis to understand the effects of biochar on the mechanical, and environmental performance of reinforced concrete.

The remainder of the paper is organized as follows: Section 2 elaborates on the methods, including the investigated materials, laboratory experiments, and finite element analysis. Section 3 presents the results from the experiments and finite element analysis. Section 4 discusses the results, and Section 5 summarizes the conclusions.

## 2. Materials and Methods

### 2.1. Overview

**Figure 1:**
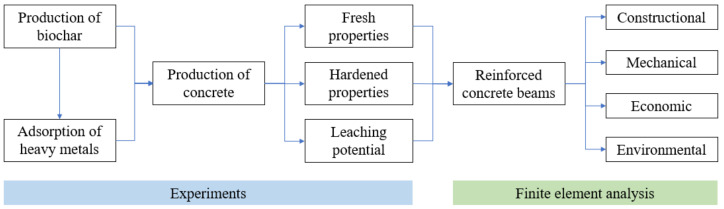
Illustration of the methods adopted in this research.

This section provides an overview of the methods, as shown in Figure 1. This research conducted laboratory experiments to evaluate the effect of biochar on the workability, compressive strengths, shrinkage, and leaching potential of concrete. Both pristine (PBC) and heavy metal-contaminated biochar (CBC) were prepared and utilized to produce concrete. The heat of cement hydration was measured to understand the underlying mechanisms of the effect of biochar on the compressive strengths of concrete. The leaching potential of concrete was evaluated through the Synthetic Precipitation Leaching Procedure (SPLP), standard Toxicity Characteristic Leaching Procedure (TCLP), and progressive TCLP. Chemical analysis was performed through inductively coupled plasma optical emission spectroscopy (ICP-OES). Based on the mechanical properties of concrete, finite element analysis was performed using a three-dimensional (3D) finite element model to investigate the effects of biochar on the mechanical properties and environmental impacts of reinforced concrete beams for structural applications. The details of the adopted methods are elaborated on in the following subsections.

### 2.2. Preparation, Characterization, and Treatment of Biochar

#### 2.2.1. Preparation

The biochar used in this research was generated from the roots of vetiver, which is a type of lignocellulosic, perennial, warm-season (or C4) grass with high efficiency at carbon sequestration for its dense root system with its roots having a high commercial value in the aromatherapy and perfumery industry [29]. After the oil is extracted, the roots are considered waste and thus disposed of. In a controlled greenhouse environment, vetiver grass was grown using slips acquired from Mosquito Hawk Farms LLC in Anahuac, TX, USA. After the roots were cleaned meticulously and air-dried for 3 days under ambient conditions, the roots were manually ground and subjected to hydro-distillation to extract the essential oil. The ground vetiver roots recovered from the oil extraction procedure were rinsed three times with deionized water to remove leftover contaminants. Using the optimized pyrolysis settings developed in a previous study [30], the roots were pyrolyzed at 500 °C for 60 min to produce biochar. A furnace (model: Lindberg 51862HR) and retort were employed to generate biochar from these feedstocks in N_2_ gas at a flow rate of 0.1 mL/min.

#### 2.2.2. Characterization of Physical and Chemical Properties

This research characterized the bulk density, yield, surface area, pH, electric conductivity, and cation exchange capacity as well as the physical and chemical properties of the biochar. The total elemental analysis for inorganics was performed following a standard method recommended by the Environmental Protection Agency (EPA) of the USA with a few minor modifications [31]. An elemental analyzer was used to determine the carbon, hydrogen, nitrogen, and oxygen elements of biochar (model: Costech 4010). Surface morphology analysis and elemental semi-quantitative analysis were performed via scanning electron microscope-energy dispersion spectroscopy (SEM-EDS) with an 80 mm^2^ silicon drift detector (Oxford Instruments, Concord, MA, USA).

The surface chemical properties were determined via Fourier transform infrared spectroscopy using an FTIR spectrometer (model: PerkinElmer Spectrum 100, Waltham, MA, USA) with spectra collected between 400 cm^−1^ and 4000 cm^−1^. Sample pellets were made for all samples using fused-KBr at a 100:1 KBr:Biochar ratio for transmission studies. An average of 100 scans at a resolution of 4 cm^−1^ were performed for each sample. The spectra were examined using commercial software, called OMNIC (v9.2.106 from Thermo Fisher Scientific Inc., Waltham, MA, USA).

#### 2.2.3. Heavy Metal Loading

The biochar was used to conduct batch adsorption tests for four heavy metals, which are Cadmium (Cd), Copper (Cu), Lead (Pb), and Zinc (Zn). These heavy metals are representative of stormwater runoff from different sources and wastewater. The solutions of Cu^2+^, Cd^2+^, Pb^2+^, and Zn^2+^ were prepared using Cu(NO_3_)_2_·3H_2_O (Fisher Scientific, Branchburg, NJ, USA), Cd(NO_3_)_2_·4H_2_O (Sigma-Aldrich, St. Louis, MO, USA), Pb(NO_3_)_2_ (Fisher Scientific, New Jersey, US), and Zn(NO_3_)_2_·6H_2_O (Fisher Scientific, New Jersey, US) salts, which were dissolved in deionized water. A solution of 0.1 M sodium nitrate (NaNO_3_) was added to the solutions to provide ionic strength. The temperature was controlled at 24 ± 2 °C, and 100 mg/L of each metal concentration was adsorbed onto 10 g biochar (1% *w*/*v*) in a 1 L solution for 24 h. Several batches were prepared to obtain the desired quantity of contaminated biochar. The initial and final concentrations of the solution were recorded. The removal percentage of heavy metals was calculated by Equation (1) [32]:

Removal percentage (%) = (C_0_ − C_e_)/(C_0_ × 100) (1)

where C_0_ and C_e_ are the initial and the final concentrations of the solution, respectively.

### 2.3. Design and Preparation of Concrete

The mixture design of concrete blended with biochar is listed in Table 1. Portland cement was used as the binder, which was partially replaced by biochar. The water-to-binder ratio was set at 0.5. River sand was utilized as fine aggregate. A high-range water reducer (HRWR) was utilized to improve the flowability. Various cement replacement percentages were considered. This research is a part of our research on ultra-high-performance concrete, which does not contain coarse aggregate. Thus, although the studied mixtures do not use coarse aggregate, they are still called concrete.

**Figure 2:**
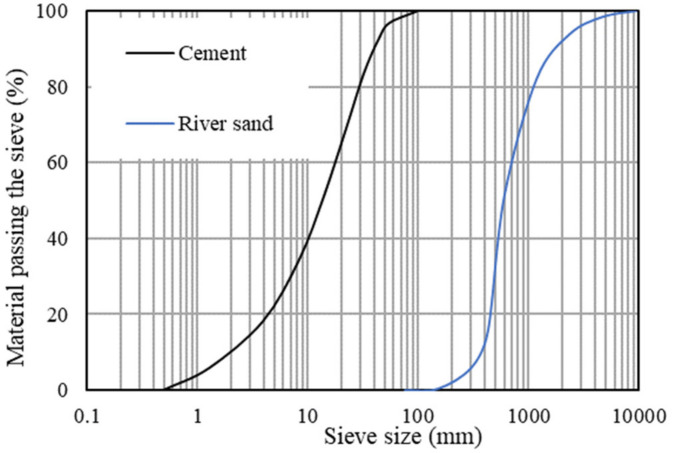
Particle size distribution of raw materials.

The particle size gradation of the cement and the sand used in the cement mixture is shown in Figure 2. The maximum grain size of the sand was 4.75 mm in diameter.

A Hobart HL200 mixer was used to prepare concrete in three steps: (1) the dry ingredients (i.e., cement, biochar, and sand) were introduced to the mixer and mixed at 107 revolutions per minute (RPM) for 2 min. (2) The HRWR was dissolved in 90% mixing water and gradually added to the mixer within 1 min. (3) The mixture was mixed at 107 RPM for 3 min.

The concrete was poured into standard molds in triplicates, including cubic molds that measure 50 mm × 50 mm × 50 mm and prism molds that measure 280 mm × 25 mm × 25 mm in dimensions. The cube specimens were used to test the compressive strengths, and the prism specimens were used to test the shrinkage. During the casting of concrete, the molds were placed on a vibrating table to ensure consolidation of concrete. Immediately after the casting, the specimens were wrapped with polythene sheets and then stored in a controlled environment at 25 °C and 70% relative humidity for 24 h. After that, the specimens were cured in lime-saturated water at 25 ± 2 °C until the date of testing. All the results characterizing the concrete performance were the mean values of triplicates. In addition, after the mixing process, all concrete mixtures were examined by hand, and no agglomeration or segregation was found.

### 2.4. Characterization of Concrete

#### 2.4.1. Workability

The workability of the concrete was evaluated according to ASTM C143 [33]. Specifically, a cone with a base diameter of 200 mm, a top diameter of 100 mm, and a height of 300 mm was used. Concrete was cast and consolidated into a cone which was then removed to let the concrete slump. The slump of fresh concrete was measured and used to evaluate the workability of concrete. Three tests were duplicated, and their results were averaged.

#### 2.4.2. Compressive Strength

The compressive strengths of concrete at 7 days and 28 days were evaluated using the cube specimens according to ASTM C109 [34]. The loading rate was kept constant at 1.8 kN/min. Three sample replicates were prepared for each test, and the average results were obtained.

#### 2.4.3. Heat of Hydration

The evolution of the heat of cement hydration was evaluated using an isothermal calorimeter (model: Calmetrix I-Cal 4000 HPC), which was programmed to maintain the samples at 25 °C. About 60 g of fresh concrete was sealed in a plastic vial and placed into the calorimeter. The measurement of the heat of hydration started 2 min after the end of mixing and continued for 36 h. The results were normalized by the mass of the cement.

#### 2.4.4. Autogenous Shrinkage

The autogenous shrinkage of concrete was evaluated in accordance with ASTM C1698 [35]. The final setting time was set as time zero. The test was conducted daily for the first week and weekly until 28 days. The prism specimens measuring 25 mm × 25 mm × 280 mm were sealed with water-proof alumina tape to prevent moisture loss. Three sample replicates were prepared for each test. The average results for each test were reported.

#### 2.4.5. Leaching Potential

The Synthetic Precipitation Leaching Procedure (SPLP) method recommended by US EPA as a batch leaching test was used to evaluate the risk of heavy metals [36]. The pH of the reagent water used in the SPLP was adjusted to 4.25 ± 0.05 using a solution of sulfuric and nitric acid at a ratio of 60:40, by volume. Then, 200 mL of the extraction fluid and 10 g of concrete were put into 500 mL high-density polyethylene (HDPE) bottles. The samples were filtered using a vacuum filtration system and a 0.6–0.8 µm glass fiber filter (Whatman GF/F) after being shaken for 18 h. After filtering, the leachate was analyzed in terms of the pH value, RCRA8 metals (As, Ba, Cd, Cr, Pb, Hg, Ag, and Se), along with the metals that were adsorbed into the biochar (Zn and Cu) by an ICP-OES.

The leachability of the trace elements from concrete to determine if it is hazardous was assessed according to the Toxicity Characteristic Leaching Procedure (TCLP) recommended by EPA [37]. A piece of concrete pellet weighing about 10 gm was obtained from the middle of the concrete block at 1 day, 14 days, and 28 days. Each sample of crushed concrete was treated for 18 h with an acetic acid solution with a pH of 2.88 ± 0.05 and a liquid-to-solid ratio of 20:1.

Progressive TCLP tests were performed to investigate the behaviors of metal pollutants that leached from the concrete [38]. There were five successive steps throughout the test. Each stage followed the same process as the typical TCLP test as mentioned above. Following each extraction, the residuals were put back into the extraction bottles so that a new batch of leachate was used. The concrete samples were transferred without first cleaning them with deionized water. The leachate was then analyzed following the analytical procedure of the SPLP protocol.

Certified reference solutions were checked beside the samples. Both internal and external standards were examined in every ICP-OES analysis. To calibrate the system, standard solutions with R^2^ > 0.995 were attained before each study. Three replicated measurements were prepared for each sample. ICP Expert Software (v7.1.0.6821) was used to operate and process the ICP-OES data (Agilent Technologies, Santa Clara, CA, USA). All the containers were cleaned with laboratory-grade detergent, steeped in 10% nitric acid solution for 12 h, soaked in deionized water for 6 h, and rinsed with deionized water. The Tukey–Kramer honestly significant difference (HSD) test was performed to assess significant differences (*p* < 0.05) using the Origin software (v2022b) [39].

### 2.5. Finite Element Analysis

**Figure 3:**
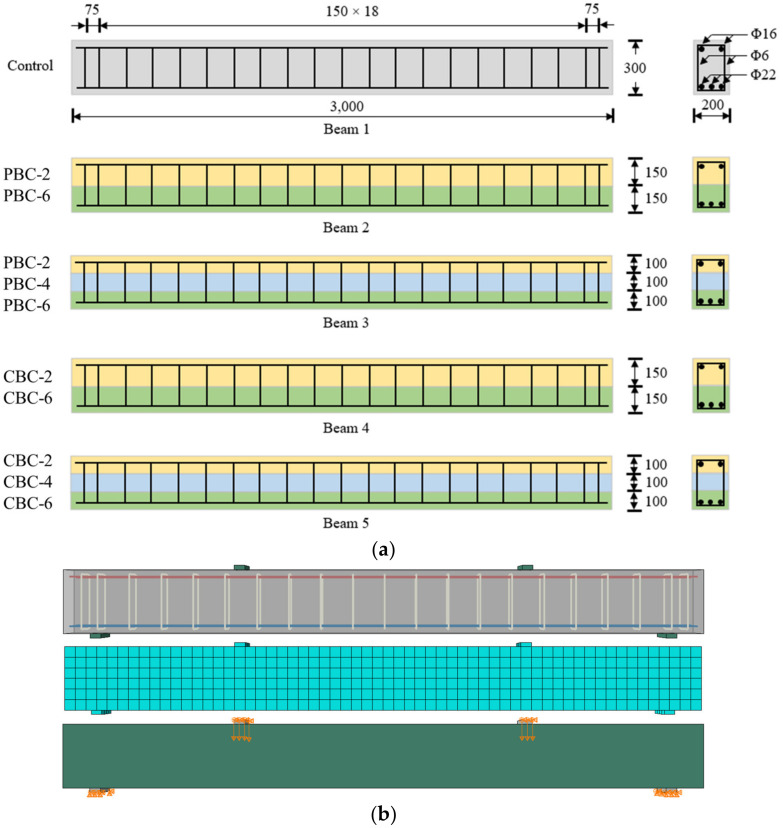
Depiction of the beams: (**a**) design and dimensions (unit: mm); (**b**) finite element model with the meshing, loading, and boundary conditions.

The flexural behaviors of reinforced concrete beams made using the concrete with biochar were investigated via 3D finite element analysis using software called ABAQUS 2017. The methods used to establish the finite element model were developed in previous research [40,41]. The cross-section of the beams was 200 mm × 300 mm, and the length of the beams was 3000 mm. Each beam was reinforced by five longitudinal steel bars and 21 stirrups. Three longitudinal bars were placed at the bottom, which was 22 mm (Φ22) in diameter and subject to tension. Two longitudinal bars were placed at the top, which was 16 mm (Φ16) in diameter and subject to compression. The diameter of the stirrups was 6 mm (Φ6). The spacing of the stirrups was 150 mm in the middle and 75 mm at the two ends of the beam. The concrete cover thickness was 20 mm. For the hybrid beam production on the job site, the authors suggest doing as follows to minimize the negative effect on the successive casting layers: (1) all designed mixtures should be mixed at the same time; (2) the workability of all mixtures should be controlled at the same level; (3) the casting interval between different concrete layers should be shortened as much as possible. The dimensions of the beams are consistent with those in the references [42,43]. Four-point loads were applied to investigate flexural behaviors. The beam design and finite element model are shown in Figure 3. The concrete and steel bars were modeled using solid elements (C3D8R) and truss elements (T3D2), respectively. The mesh size of the concrete and steel bars was determined through a mesh sensitivity analysis. The global mesh sizes of the concrete beam and steel bars were 5 mm and 10 mm, respectively.

The boundary conditions of the beams were defined as follows: (1) in the symmetrical plane at the mid-span section, the displacement along the length direction is zero. (2) In the symmetrical plane at the mid-width section, the displacement along the width direction is zero. (3) Along the lines over the rollers, the displacement along the vertical direction is zero. The vertical loads were applied to the beam under the displacement control mode. The study utilized the embedded region constraint in the software ABAQUS 2017 to model the interaction between concrete and steel reinforcement by using the keyword “embed”, meaning that debonding was not considered. This method has been widely employed in finite element analysis on the behavior of reinforced concrete structures [44,45,46,47]. The model with the embedded region constraint does not need the input of the material properties of concrete and steel reinforcement.

The density and Poisson’s ratio of concrete were 2600 kg/m^3^ and 0.2, respectively. The density and Poisson’s ratio of steel bars were 7800 kg/m^3^ and 0.3, respectively. The yielding strengths of the steel bars were related to their diameters. The yielding strengths of the steel bars with diameters 6 mm, 16 mm, and 22 mm were 420 MPa, 445 MPa, and 430 MPa, respectively. The elastic modulus of steel bars was 205 GPa. These material properties are consistent with previous research on experiments and finite element analysis of the flexural behaviors of reinforced concrete [42]. The compressive strength of concrete is not listed because the value was obtained from experiments, as elaborated in Section 2.2. The elastic modulus of concrete was calculated according to ACI 318 [48].

**Figure 4:**
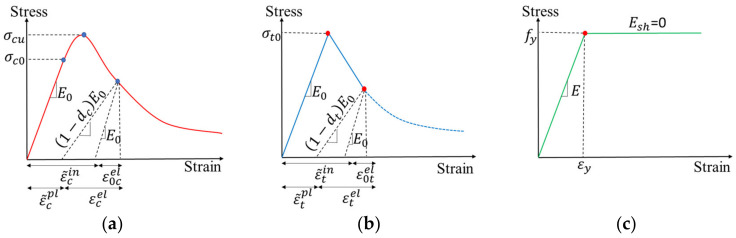
Illustration of the constitutive relationships of (**a**) concrete in compression; (**b**) concrete in tension; and (**c**) steel bars.

The concrete damage plasticity model was applied to consider the damage in concrete during the loading process. More detailed information about the concrete damage plasticity model is available in references [49,50] and is replicated in this paper. The constitutive relationship of concrete is associated with compressive strength, as shown in Figure 4a. The parameters of the adopted concrete damage plasticity model are listed in Table 2. The elastic-perfectly-plastic constitutive model was employed in this study, as it is a common and simplified model to describe steel behavior [51,52], as shown in Figure 4b.

## 3. Results

### 3.1. Characteristics of Biochar

The physicochemical characteristics of the biochar are listed in Table 3. The yield of the vetiver root biochar was 53.76% with a surface area of 308.15 m^2^/g. The pH was about 11. This biochar had a high carbon concentration. According to the acid digestion method [31], the biochar also had trace amounts of inorganic components likely due to the presence of the engineered soil used to grow the plants in the greenhouse. The molar H/C, O/C, and N/C ratios were 0.04, 0.25, and 0.3, respectively. The H/C and O/C ratios indicate that the biochar is more aromatic than polar. Previous research also indicated that the high pH and high liming value of this biochar make it a potential liming agent for the reclamation of acid mine drainage-impacted soils [30].

**Figure 5:**
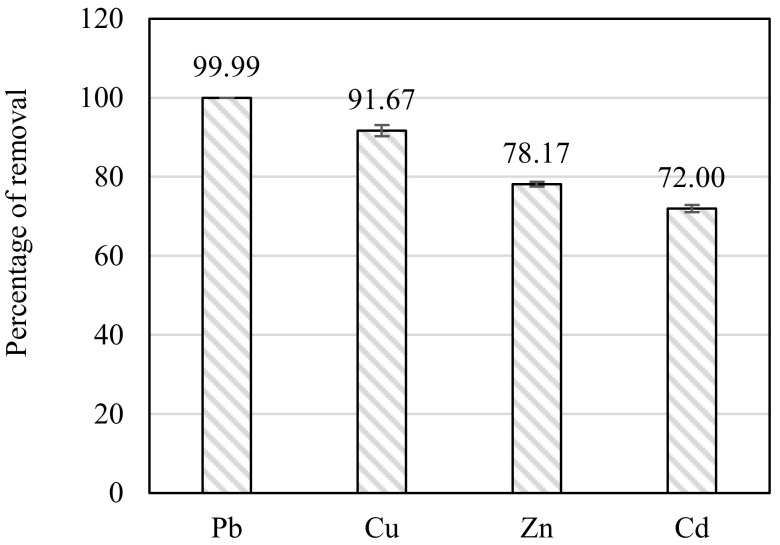
Results of the removal percentage of the heavy metals using biochar.

The efficiency of using biochar in removing heavy metals through multi-metal batch adsorption experiments is shown in Figure 5. In the adsorption experiments, 100 mg/L of each metal was used to react with the biochar. After 24 h of reaction, the percentage of the removal of heavy metals was 99.99% for Pb, 91.67% for Cu, 78.17% for Zn, and 72.00% for Cu. The contaminated biochar was compared with pristine biochar in producing concrete.

### 3.2. Characteristics of Concrete

#### 3.2.1. Workability

**Figure 6:**
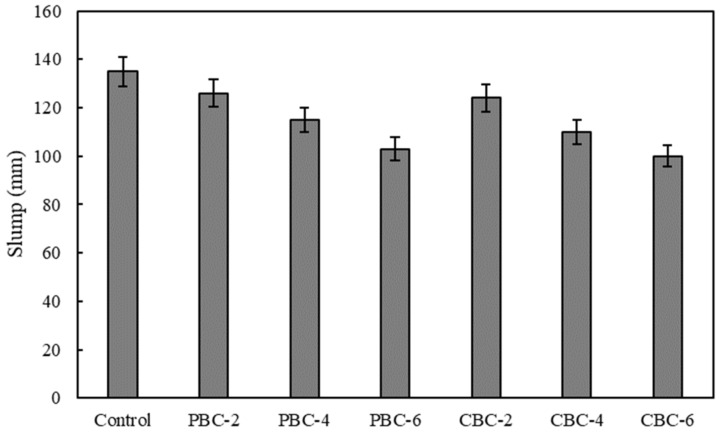
Results of the slump for evaluating the workability of the concrete mixtures.

The effect of biochar contents on the workability of fresh concrete mixtures is shown in Figure 6. When the HRWR content was the same in all the investigated concrete mixtures, the workability of concrete decreased with the increase of the biochar content, meaning that the use of biochar compromises the workability of concrete. Such results are consistent with the findings from prior research [53,54,55,56]. The main reason for the reduction of the workability of concrete is that the porous biochar particles have a large specific surface area and can absorb the mixing water when they are not pre-soaked.

In this research, the biochar was not pre-soaked because pre-soaking biochar with additional water would increase the total water content and, thus, the water-to-cement ratio of the concrete mixtures. It is known that the increase in the water-to-cement ratio tends to reduce the mechanical strength and durability of concrete. In practice, if the workability is sufficient for construction, it is often effective to increase the HRWR content [57]. The test results also show that the presence of heavy metals adsorbed by biochar does not affect the workability of concrete.

#### 3.2.2. Compressive Strengths

**Figure 7:**
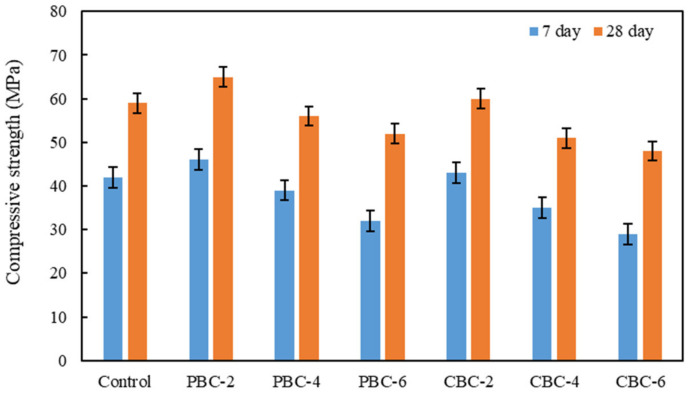
Results of the compressive strengths of the mixtures at 7 days and 28 days.

The compressive strengths of the concrete mixtures at 7 days and 28 days are shown in Figure 7. When pristine biochar was used, with the biochar content varying from 0 to 6%, the compressive strengths of concrete at 7 days and 28 days followed the same trend: The compressive strengths first increased and then decreased. The highest compressive strengths were achieved when the pristine biochar content was 2%.

Such results are caused by two competing effects. On one hand, biochar provides filler effects to increase the compressive strength of concrete. On the other hand, biochar introduces defects such as pores and interfacial transition zones to decrease the compressive strength of concrete. When the biochar content was lower than 2%, the filler effect was dominant. When the biochar content was higher than 2%, the weakening effects became dominant. These results are consistent with previous research [15,58]. The results show that the heavy metals in the biochar reduce the compressive strength of concrete. When the contaminated biochar content was 2%, the compressive strengths at 7 days and 28 days were comparable with those of the control concrete.

#### 3.2.3. Heat of Hydration

**Figure 8:**
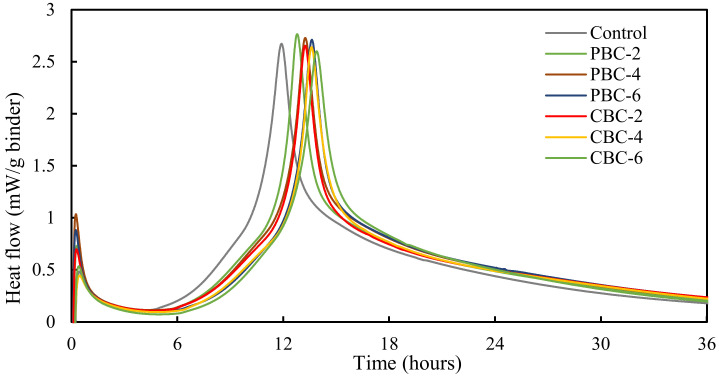
Results of the heat of hydration normalized by the mass of cement.

The results of the heat of hydration are shown in Figure 8. When the pristine biochar was used, the biochar content increased from 0 to 6%. The peak heat flow was delayed from 11.9 h to 13.3 h. With the increase in contaminated biochar from 0 to 6%, the peak heat flow was delayed from 11.9 h to 14.0 h. These results reveal that the use of biochar retarded the hydration reactions of cement.

According to reference [59], the hydration kinetics of cement paste, mortar, and concrete is related to the presence of impurities and saccharides in biochar and the pretreatment of biochar. The addition of biochar may either accelerate or delay the hydration of cement. For instance, the biochar prepared from sorghum retarded the hydration heat (approximately 2 h) and decreased the peak magnitude [59]. The underlying reason is that the pyrolyzing sorghum at 500 °C decomposes the cellulose and hemicellulose, which are the key components that hinder the hydration of cement [60]. In addition, Gupta et al. [54] found that, compared with the cement paste with dry biochar, the addition of pre-soaked biochar further promoted the hydration of cement because the pre-soaked biochar released extra water to the cement matrix.

In this study, the roots were pyrolyzed at 500 °C for 60 min to produce biochar. The cellulose and hemicellulose were decomposed during the production process to retard the cement hydration. Moreover, the dry biochar powder without pre-soak treatment was directly used in this study. Since the mechanical strengths of concrete are gained through the hydration reactions, the results of the heat of hydration explain why the excessive addition of biochar reduced the compressive strengths of concrete. The results also show that the presence of heavy metals further retarded the hydration reactions of cement, thus further reducing the compressive strengths of concrete.

#### 3.2.4. Autogenous Shrinkage

**Figure 9:**
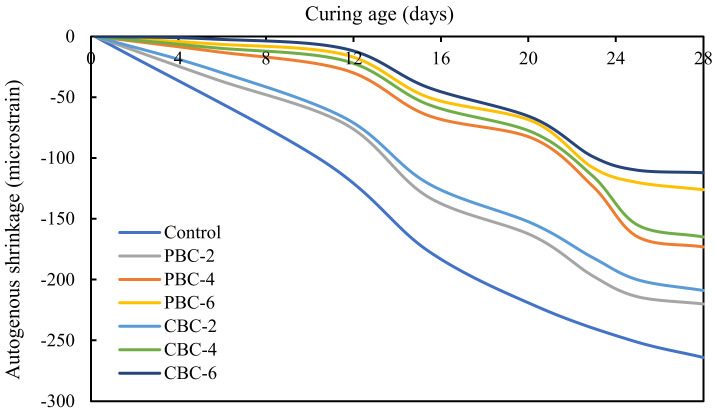
Results of the autogenous shrinkage of the concrete mixtures at different ages.

The results of the autogenous shrinkage of the investigated concrete mixtures are shown in Figure 9. The use of biochar efficiently reduced the autogenous shrinkage of the concrete. With the increase in pristine biochar content from 0 to 6%, the autogenous shrinkage at 28 days was reduced from 264 µε to 126 µε (by 50%). With the increase in contaminated biochar content from 0 to 6%, the autogenous shrinkage at 28 days was reduced from 264 µε to 112 µε (by 58%).

There are two main underlying mechanisms: (1) porous biochar particles absorbed water and released the internal curing water during the hardening process to delay the drop of the internal relative humidity inside the concrete, thus reducing the autogenous shrinkage. (2) The biochar retarded the hydration of cement, thus alleviating the autogenous shrinkage.

### 3.3. Leaching Potential

The concentrations of the heavy metals in the leachates from the SPLP test are listed in Table S1 in the Supplementary Material. The concentrations of the heavy metals are evaluated in accordance with US EPA requirements. The concentrations of Hg, As, Se, Pb, Cd, Ag, and Ba are far below the EPA limits. EPA has not set limits for Cu and Zn. Many of the results were below the detection limit, suggesting that the leaching potential of the biochar and concrete was low. In other words, there is no concern about the leaching of heavy metals when contaminated biochar is used to produce concrete.

The concentrations of the heavy metals in the leachates from the standard TCLP test are listed in Table S2. The values of the concentrations of the heavy metals are different from those from the SPLP test but are consistent with the observations that the concentrations of the heavy metals are far below the EPA limits. Many of the results were below the detection limit, corroborating that there is no concern about the leaching of the heavy metals when the contaminated biochar is utilized to produce concrete.

The concentrations of the heavy metals of the progressive TCLP test are listed in Tables S3–S5. Again, the results are consistent with the results from the standard TCLP test. The progressive TCLP test further verifies that the concentrations of the heavy metals are far below the EPA limits. There were incremental increases in the concentrations of the heavy metals as the test continued progressively with time. However, the low concentrations show that such concrete will not leach out heavy metal contamination despite being in contact with acidic environmental factors such as acid rain.

**Figure 10:**
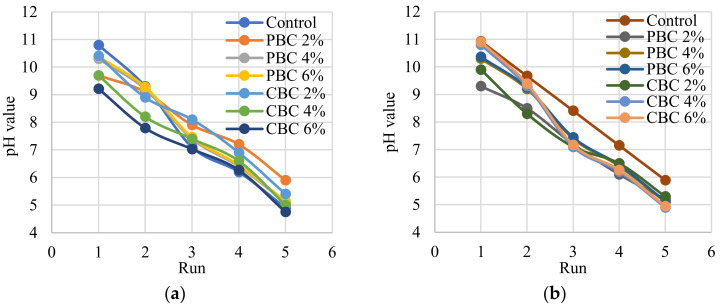

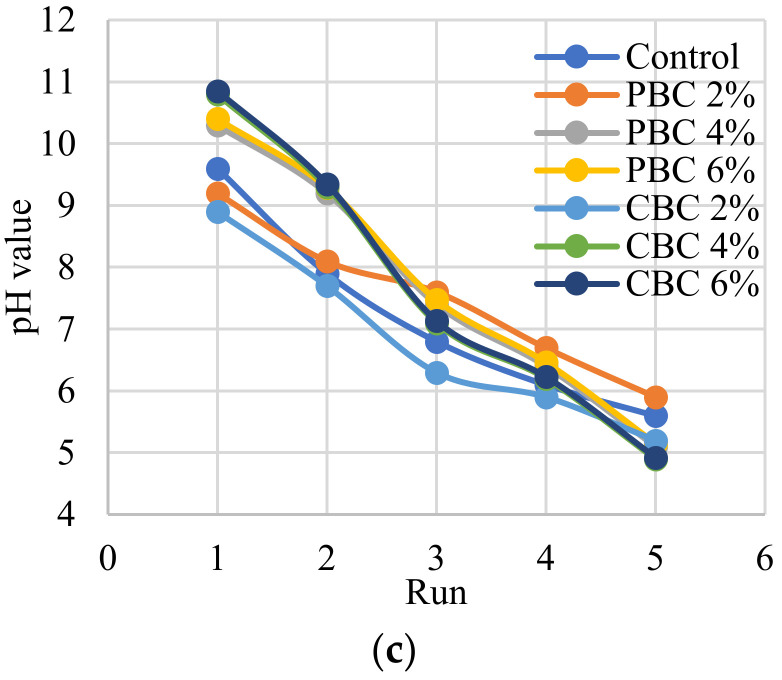
Results of the pH values in the progressive TCLP tests of the different concrete mixtures at different ages: (**a**) 1 day, (**b**) 14 days, and (**c**) 28 days.

The pH values of the leachate in the progressive TCLP test are plotted in Figure 10. The pH values at the completion of each treatment were much higher than the pH value of the original extraction solution, which was 2.88. The increase in the pH value can be attributed to the high pH of the concrete. The pH value dropped as the TCLP extraction progressed, indicating that the biochar can function as a buffer. The pH values are not very sensitive to the change in the biochar content or the change in the concrete age.

### 3.4. Finite Element Analysis

**Figure 11:**
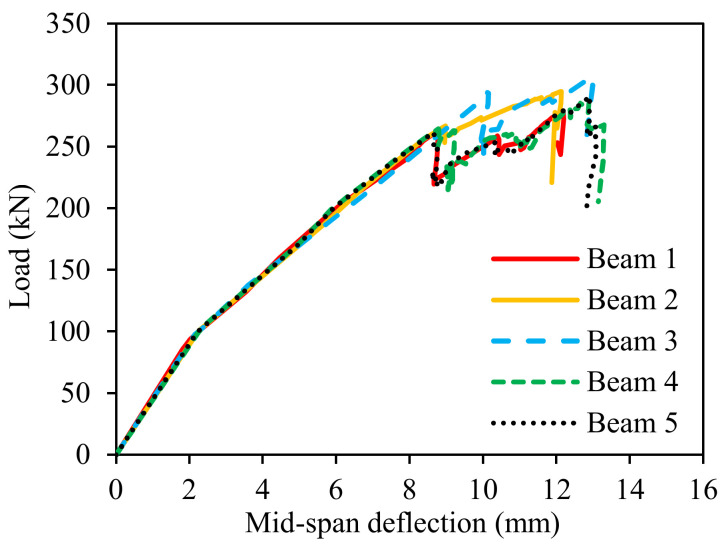
Results of the load–displacement curves of the five reinforced concrete beams.

Figure 11 shows the load–displacement curves obtained from finite element analysis. The load increased linearly with displacement until the first crack in the concrete appeared. Then, the slope of the curves decreased due to the development of the cracks in concrete. The beams could resist a higher load after the concrete was cracked because of the use of steel bars.

The differences in peak load between Beams 1, 4, and 5 were small. For instance, the difference in peak load between Beams 4 and 5 was 4 MPa. Given the vertical axis in Figure 11, the difference between the peak loads is unclear. A clearer representation of the difference in peak load and energy dissipation is given in Section 4.1.

Previous studies revealed that the biochar addition does not significantly alter the modulus of elasticity of mortar [58,61]. For instance, an addition of 8% biochar (by mass) reduced the elastic modulus of mortar by approximately 10%. This study mainly investigated the amendment of cement mortar with different biochar ratios. Without the fiber reinforcement, the cement mortar would show brittle rupture. In this case, the tensile behaviors of the investigated cement mortar were not satisfactory. Thus, further experimental tests to characterize the tensile behavior of cement mortar with different biochar contents in this study were not conducted.

**Figure 12:**
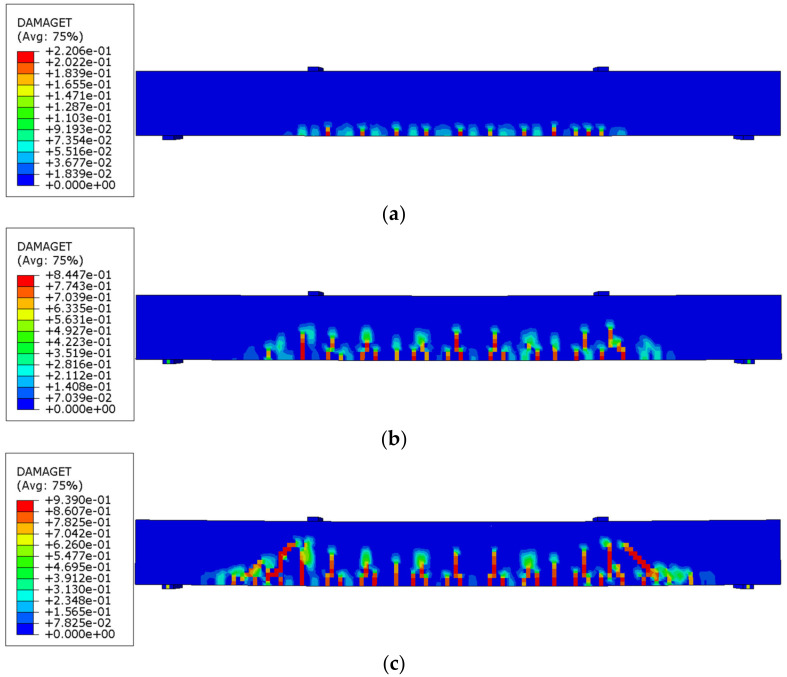
Visualization of the development of cracks in Beam 3 under increasing displacements: (**a**) 1.34 mm, (**b**) 5.53 mm, and (**c**) 10.22 mm.

The cracks in the concrete are quantitatively represented by a tension damage index, which is denoted by DAMAGET. DAMAGET is in the range of 0 to 1: 0 means there is no tension damage (i.e., crack), and 1 means the concrete is completely cracked. Figure 12 shows the finite element analysis results of the development of cracks in Beam 3 with the increase of the displacement applied to the beam. With the increase of the displacement, the cracks in concrete are initiated from the mid-span section and then developed toward the two ends of the beam. The cracks are mainly flexural cracks located within the flexural span. After the flexural cracks were developed to a certain level, diagonal shear cracks were generated in the shear spans.

## 4. Discussion

### 4.1. Constructional and Mechanical Performance

The workability of concrete is relevant to the construction quality as poor workability often causes problems with the placement and consolidation of concrete in the construction of structures such as bridges and buildings [62]. The reason is that poor consolidation in turn produces voids, which compromise not only the mechanical properties such as the compressive strengths but also the durability of concrete. Figure 6 shows that as the biochar content increases from 0 to 6%, the slump of concrete decreases from 135 mm to 103 mm. The reduction of the slump adversely affects the construction quality of the reinforced concrete beams. Therefore, in structural applications, it might be necessary to use a water reducer to increase the workability of concrete with biochar.

**Figure 13:**
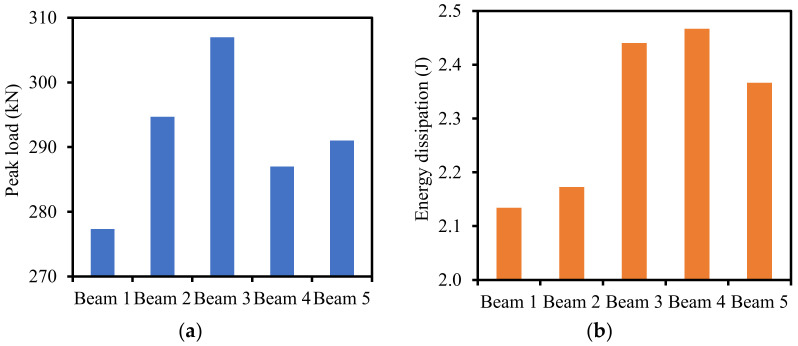
The comparison of the flexural behaviors of the investigated beams: (**a**) peak load, and (**b**) energy dissipation.

The results of finite element analysis show that utilizing biochar can improve the mechanical performance of the investigated beams. Figure 13a shows the load capacity results of the five reinforced concrete beams. The use of pristine and contaminated biochar improved the flexural performance of the reinforced concrete beams. The load capacities of Beam 2, Beam 3, Beam 4, and Beam 5 increased by 6%, 11%, 3%, and 5%, respectively, compared with the load capacity of Beam 1. The load capacity of Beam 3 with PBC-2, PBC-4, and PBC-6 is 11% higher than that of Beam 1.

The results of energy dissipation of the investigated beams are shown in Figure 13b. Energy dissipation was calculated by the area under the load–deflection curve. The use of biochar in Beam 2, Beam 3, Beam 4, and Beam 5 increased the energy dissipation by 7%, 14% 16%, and 14%, respectively, compared with Beam 1.

### 4.2. Economic and Environmental Performance

The economic and environmental performance of the beams were evaluated using the data in Table 4. The unit material cost and unit carbon emission of the different ingredients were reported in previous research [63,64]. Biochar was regarded as a waste material [65]. The material cost and carbon emission of each beam were calculated by Equation (2) and Equation (3), respectively:

(2) $$M=\sum_{i=1}^{n}m_{i}r_{i}$$

where *M* is the unit cost of a concrete mixture per cubic meter (unit: $/m^3^); *m_i_* is the unit cost (unit: $/kg) of the *i*th ingredient of the mixture (*i* = 1, 2, 3, …, *n*, and *n* = 5), as listed in Table 4; and *r_i_* is the mass of the *i*th ingredient of the mixture (unit: kg/m^3^), as listed in Table 1.

(3) $$C=\sum_{i=1}^{n}c_{i}r_{i}$$

where *C* is the carbon footprint of a concrete mixture; *c_i_* is the unit carbon emission of the *i*th ingredient of the mixture (*i* = 1, 2, 3, …, *n*, and *n* = 5), as listed in Table 4; and *r_i_* is the mass of the *i*th ingredient of the mixture, as listed in Table 1.

**Figure 14:**
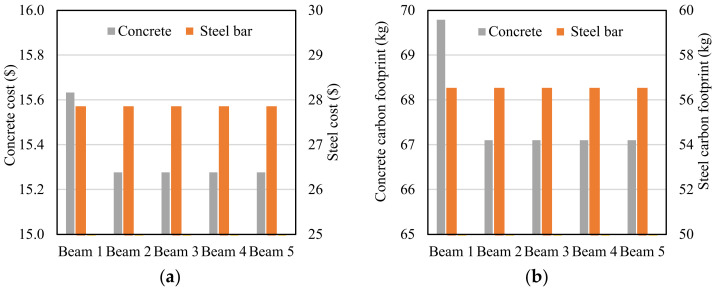
Results of the cost and carbon footprint of the reinforced concrete beams: (**a**) cost and (**b**) carbon footprint.

The calculation results are plotted in Figure 14. The results show that the use of biochar in the concrete reduces the cost and the carbon footprint of the beams. When the beam is made of multiple layers of concrete with different biochar contents, the cost and the carbon footprint of the beam can be further reduced. Beam 3 and beam 5 achieved the lowest cost and lowest carbon footprint.

## 5. Conclusions

This study investigates the effects of using biochar in low-cost low-carbon concrete on the mechanical, economic, and environmental performance of reinforced concrete beams through comprehensive laboratory experiments of the concrete and finite element analysis of the reinforced concrete beams. The following conclusions can be drawn from the above investigations:

- The use of biochar was able to increase the compressive strength of concrete. When biochar was used to replace 2% of Portland cement in the concrete, the compressive strengths of the concrete were increased. When the replacement percentage was higher than 2%, the use of biochar reduced the compressive strengths of concrete. Compared with pristine biochar, the contamination of biochar by heavy metals reduced the compressive strengths due to the delay of hydration of cement.
- The use of biochar decreased the shrinkage of concrete. When the biochar was used to replace 6% of Portland cement in the concrete, the autogenous shrinkage of concrete was reduced from 264 µε to 112 µε. The main mechanism was the internal curing effect of porous biochar. Compared with pristine biochar, the contamination of biochar by heavy metals reduced the autogenous shrinkage due to the delay of hydration of cement.
- The use of biochar decreased the workability of concrete. When biochar was used to replace 6% of Portland cement in the concrete, the slump of the concrete was reduced from 135 mm to 103 mm. The main mechanism was the water absorption effect of porous biochar. Compared with pristine biochar, the contamination of biochar by heavy metals did not affect the workability of concrete.
- The use of biochar in concrete did not cause a leaching problem. The concentrations of the heavy metals were well below the EPA limits, indicating that the biochar that was used to absorb heavy metals was not a hazardous waste. In other words, it is promising to use biochar to immobilize the heavy metals and then produce cost-effective eco-friendly concrete.
- The use of concrete blended with biochar was able to improve the mechanical properties, economic, and environmental performance of reinforced concrete beams. Using concrete with different biochar contents in layers was able to further improve the economic and environmental performance of reinforced concrete beams while retaining the flexural properties of the beams.

Future research is needed to understand the influence of contaminated biochar on the durability of concrete and reinforced concrete beams. It is also important to evaluate the effect of biochar on the demand for water reducers to ensure adequate flowability and constructional performance.

## Figures and Tables

**Table 1:** Mixture design of concrete with biochar (kg/m^3^).

Designation	Cement	PBC	CBC	Sand	HRWR	Water
Control	450	0	0	1350	1.0	225
PBC-2	441	9	0	1350	1.0	225
PBC-4	432	18	0	1350	1.0	225
PBC-6	423	27	0	1350	1.0	225
CBC-2	441	0	9	1350	1.0	225
CBC-4	432	0	18	1350	1.0	225
CBC-6	423	0	27	1350	1.0	225

Note: PBC-2 represents concrete with 2% cement replaced by pristine biochar, by mass. CBC-6 represents concrete with 6% cement replaced by contaminated biochar, by mass.

**Table 2:** Parameters used in the CDP model.

Parameter	Dilation Angle	Eccentricity Parameter	Viscosity Parameter	Shape of the Yielding Surface	Stress Ratio
Value	36°	0.1	0.001	0.667	1.05

**Table 3:** Physicochemical characteristics of biochar.

Parameters	Results
Pyrolysis temperature	500 °C
Pyrolysis time	60 min
Yield	53.76%
BET surface area	308.2 m^2^/g
pH value	10.98 ± 0.11
Electric conductivity	162.07 ± 13.13 µS/cm
Ash content	26.4%
Cation exchange capacity	82 cmol/kg
Bulk density	0.62 gm/mL
Carbon	68.58%
Hydrogen	2.78%
Oxygen	1.75%
Nitrogen	20.7%
Hydrogen/Carbon	0.041
Oxygen/Carbon	0.026
Nitrogen/Carbon	0.302

**Table 4:** Inventory data of unit material cost and unit carbon emission.

Material	Unit Cost ($/kg)	Unit Carbon Emission (kg/kg)
Portland cement	0.11 [63]	0.83 [66]
Biochar	0 [67]	0 [67]
River sand	0.02 [68]	0.01 [69]
HRWR	3.60 [70]	0.72 [64]
Water	0 [71]	0 [66]
Steel rebar	0.68 [72]	1.38 [73]

## Data Availability

Data presented in this study are available on request from the corresponding author.

## Supplementary Materials

The following supporting information can be downloaded at: https://www.mdpi.com/article/10.3390/ma16062522/s1, Table S1: Results of the SPLP test (unit: µg/L). Table S2: Results of the TCLP test (unit: µg/L). Table S3: Progressive TCLP data at 1 day (unit: µg/L). Table S4: Progressive TCLP data at 14 days (unit: µg/L). Table S5: Progressive TCLP data at 28 days (unit: µg/L).

## References

[1] Barcelo L., Kline J., Walenta G., Gartner E. (2013). Cement and carbon emissions. Mater. Struct..

[2] Andrew R.M. (2019). Global CO_2_ emissions from cement production, 1928–2018. Earth Syst. Sci. Data.

[3] Meng W., Valipour M., Khayat K.H. (2017). Optimization and performance of cost-effective ultra-high performance concrete. Mater. Struct..

[4] Du J., Liu Z., Christodoulatos C., Conway M., Bao Y., Meng W. (2022). Utilization of off-specification fly ash in preparing ultra-high-performance concrete (UHPC): Mixture design, characterization, and life-cycle assessment. Resour. Conserv. Recycl..

[5] Li X., Lv X., Zhou X., Meng W., Bao Y. (2021). Upcycling of waste concrete in eco-friendly strain-hardening cementitious composites: Mixture design, structural performance, and life-cycle assessment. J. Clean. Prod..

[6] Wong C.L., Mo K.H., Yap S.P., Alengaram U.J., Ling T.-C. (2018). Potential use of brick waste as alternate concrete-making materials: A review. J. Clean. Prod..

[7] Guo P., Meng W., Nassif H., Gou H., Bao Y. (2020). New perspectives on recycling waste glass in manufacturing concrete for sustainable civil infrastructure. Constr. Build. Mater..

[8] Sharma R., Bansal P.P. (2016). Use of different forms of waste plastic in concrete—A review. J. Clean. Prod..

[9] Mahjoubi S., Barhemat R., Meng W., Bao Y. (2023). AI-guided auto-discovery of low-carbon cost-effective ultra-high performance concrete (UHPC). Resour. Conserv. Recycl..

[10] Du J., Meng W., Khayat K.H., Bao Y., Guo P., Lyu Z., Abu-Obeidah A., Nassif H., Wang H. (2021). New development of ultra-high-performance concrete (UHPC). Compos. Part B Eng..

[11] Spiesz P., Rouvas S., Brouwers H. (2016). Utilization of waste glass in translucent and photocatalytic concrete. Constr. Build. Mater..

[12] Torres A., Bartlett L., Pilgrim C. (2017). Effect of foundry waste on the mechanical properties of Portland Cement Concrete. Constr. Build. Mater..

[13] Ling T.C., Nor H.M. Granulated waste tyres in concrete paving block. Proceedings of the 6th Asia-Pacific Structural Engineering and Construction Conference.

[14] Modarres A., Hesami S., Soltaninejad M., Madani H. (2016). Application of coal waste in sustainable roller compacted concrete pavement-environmental and technical assessment. Int. J. Pavement Eng..

[15] Gupta S., Kua H.W., Koh H.J. (2018). Application of biochar from food and wood waste as green admixture for cement mortar. Sci. Total Environ..

[16] Khushnood R.A., Ahmad S., Restuccia L., Spoto C., Jagdale P.V., Tulliani J.-M., Ferro G.A. (2016). Carbonized nano/microparticles for enhanced mechanical properties and electromagnetic interference shielding of cementitious materials. Front. Struct. Civ. Eng..

[17] Gupta S., Kua H.W., Pang S.D. (2019). Effect of biochar on mechanical and permeability properties of concrete exposed to elevated temperature. Constr. Build. Mater..

[18] Tan K., Qin Y., Wang J. (2022). Evaluation of the properties and carbon sequestration potential of biochar-modified pervious concrete. Constr. Build. Mater..

[19] Tan K., Wang J. (2023). Substrate modified with biochar improves the hydrothermal properties of green roofs. Environ. Res..

[20] Aziz M.A., Zubair M., Saleem M., Alharthi Y.M., Ashraf N., Alotaibi K.S., Aga O., Al Eid A.A.A. (2023). Mechanical, non-destructive, and thermal characterization of biochar-based mortar composite. Biomass Convers. Biorefinery.

[21] Mo L., Fang J., Huang B., Wang A., Deng M. (2019). Combined effects of biochar and MgO expansive additive on the autogenous shrinkage, internal relative humidity and compressive strength of cement pastes. Constr. Build. Mater..

[22] Meng W., Khayat K. (2017). Effects of saturated lightweight sand content on key characteristics of ultra-high-performance concrete. Cem. Concr. Res..

[23] Lyu Z., Shen A., Meng W. (2021). Properties, mechanism, and optimization of superabsorbent polymers and basalt fibers modified cementitious composite. Constr. Build. Mater..

[24] Chen L., Zhang Y., Wang L., Ruan S., Chen J., Li H., Yang J., Mechtcherine V., Tsang D.C. (2021). Biochar-augmented carbon-negative concrete. Chem. Eng. J..

[25] Wang L., Chen L., Tsang D.C., Guo B., Yang J., Shen Z., Hou D., Ok Y.S., Poon C.S. (2020). Biochar as green additives in cement-based composites with carbon dioxide curing. J. Clean. Prod..

[26] Godwin P.M., Pan Y., Xiao H., Afzal M.T. (2019). Progress in preparation and application of modified biochar for improving heavy metal ion removal from wastewater. J. Bioresour. Bioprod..

[27] Wang L., Wang Y., Ma F., Tankpa V., Bai S., Guo X., Wang X. (2019). Mechanisms and reutilization of modified biochar used for removal of heavy metals from wastewater: A review. Sci. Total Environ..

[28] Chen Q.Y., Tyrer M., Hills C.D., Yang X.M., Carey P. (2009). Immobilisation of heavy metal in cement-based solidification/stabilisation: A review. Waste Manag..

[29] Neve S., Sarkar D., Zhang Z., Datta R. (2022). Optimized Production of Second-Generation Bioethanol from a Spent C4 Grass: Vetiver (*Chrysopogon zizanioides*). Energies.

[30] Neve S., Sarkar D., Datta R. Effects of Pyrolysis Temperature and Residence Time on Physicochemical Properties of Biochar Derived from Spent Vetiver Roots. Proceedings of the ASA, CSSA, SSSA International Annual Meeting.

[31] Kimbrough D.E., Wakakuwa J.R. (1989). Acid digestion for sediments, sludges, soils, and solid wastes. A proposed alternative to EPA SW 846 Method 3050. Environ. Sci. Technol..

[32] Feng J., Yang Z., Zeng G., Huang J., Xu H., Zhang Y., Wei S., Wang L. (2013). The adsorption behavior and mechanism investigation of Pb(II) removal by flocculation using microbial flocculant GA1. Bioresour. Technol..

[33] (2020). Standard Test Method for Slump of Hydraulic-Cement Concrete.

[34] (2021). Standard Test Method for Compressive Strength of Hydraulic Cement Mortars.

[35] (2020). Standard Test Method for Autogenous Strain of Cement Paste and Mortar.

[36] Montour M.R., Hageman P.L., Meier A.L., Theodorakos P., Briggs P.H. (2022). EPA Method 1312 (Synthetic Precipitation Leaching Procedure); Leachate Chemistry Data for Solid Mine Waste Composite Samples from Silverton and Leadville, Colorado.

[37] EPA, U. S. (1992). Toxicity Characteristics Leaching Procedure, Method 1311. Test Methods for the Evaluation of Solid Waste.

[38] Li X.D., Poon C.S., Sun H., Lo I.M.C., Kirk D.W. (2001). Heavy metal speciation and leaching behaviors in cement based solidified/stabilized waste materials. J. Hazard. Mater..

[39] (2022). OriginPro, Version 2022b.

[40] Liu Y., Bao Y., Deng L., Zhang Q. (2023). Experimental and finite element investigations on shear behaviors of stud connectors embedded in Engineered Cementitious Composite (ECC). Eng. Struct..

[41] Meng W., Khayat K.H. (2016). Experimental and numerical studies on flexural behavior of ultrahigh-performance concrete panels reinforced with embedded glass fiber-reinforced polymer grids. Transp. Res. Rec..

[42] Li X., Lu X., Qi J., Bao Y. (2022). Flexural behavior of fire-damaged concrete beams repaired with strain-hardening cementitious composite. Eng. Struct..

[43] Li X., Li Y., Yan M., Meng W., Lu X., Chen K., Bao Y. (2021). Cyclic behavior of joints assembled using prefabricated beams and columns with Engineered Cementitious Composite (ECC). Eng. Struct..

[44] ABAQUS/CAE (2017). Software for Technical Computation.

[45] Vellaipandian K., Mydeen M.S.S.M., Periasamy R.P., Soosaimarian J.J. (2023). Effects of metakaolin on the shear capacity of EBFRP RC beams: An experimental and numerical investigation. Constr. Build. Mater..

[46] Obaidat A.T. (2021). Flexural behavior of reinforced concrete beam using CFRP hybrid system. Eur. J. Environ. Civ. Eng..

[47] Long X., Lee C. (2015). Modelling of Two Dimensional Reinforced Concrete Beam-Column Joints Subjected to Monotonic Loading. Adv. Struct. Eng..

[48] (2019). Building Code Requirements for Structural Concrete (ACI 318-19) and Commentary.

[49] Sumer Y., Aktaş M. (2015). Defining parameters for concrete damage plasticity model. Chall. J. Struct. Mech..

[50] Hafezolghorani M., Hejazi F., Vaghei R., Jaafar M.S.B., Karimzade K. (2017). Simplified Damage Plasticity Model for Concrete. Struct. Eng. Int..

[51] Salmon C.G., Johnson J.E., Malhas F.A. (2008). Steel Structures Design and Behavior.

[52] Asadnia M., Roddis W.K. Modeling out-of-flatness and residual stresses in steel plate girders. Proceedings of the Annual Stability Conference Structural Stability Research Council.

[53] Choi W.C., Yun H.D., Lee J.Y. (2012). Mechanical properties of mortar containing bio-char from pyrolysis. J. Korea Inst. Struct. Maint. Insp..

[54] Gupta S., Kua H.W. (2018). Effect of water entrainment by pre-soaked biochar particles on strength and permeability of cement mortar. Constr. Build. Mater..

[55] Muthukrishnan S., Gupta S., Kua H.W. (2019). Application of rice husk biochar and thermally treated low silica rice husk ash to improve physical properties of cement mortar. Theor. Appl. Fract. Mech..

[56] Sirico A., Bernardi P., Belletti B., Malcevschi A., Dalcanale E., Domenichelli I., Fornoni P., Moretti E. (2020). Mechanical characterization of cement-based materials containing biochar from gasification. Constr. Build. Mater..

[57] Meng W., Khayat K.H. (2018). Effect of hybrid fibers on fresh properties, mechanical properties, and autogenous shrink-age of cost-effective UHPC. J. Mater. Civ. Eng..

[58] Maljaee H., Madadi R., Paiva H., Tarelho L., Ferreira V.M. (2021). Incorporation of biochar in cementitious materials: A roadmap of biochar selection. Constr. Build. Mater..

[59] Tan K.H., Wang T.Y., Zhou Z.H., Qin Y.H. (2021). Biochar as a partial cement replacement material for developing sustainable concrete: An overview. J. Mater. Civ. Eng..

[60] Gupta S., Kua H.W. (2020). Combination of biochar and silica fume as partial cement replacement in mortar: Performance evaluation under normal and elevated temperature. Waste Biomass Valoriz..

[61] Gupta S., Kua H.W., Pang S.D. (2018). Biochar-mortar composite: Manufacturing, evaluation of physical properties and economic viability. Constr. Build. Mater..

[62] Tattersall G.H. (1991). Workability and Quality Control of Concrete.

[63] Wille K., Boisvert-Cotulio C. (2015). Material efficiency in the design of ultra-high performance concrete. Constr. Build. Mater..

[64] Chiaia B., Fantilli A.P., Guerini A., Volpatti G., Zampini D. (2014). Eco-mechanical index for structural concrete. Constr. Build. Mater..

[65] Dixit A., Gupta S., Pang S.D., Kua H.W. (2019). Waste Valorisation using biochar for cement replacement and internal curing in ultra-high performance concrete. J. Clean. Prod..

[66] Long G., Gao Y., Xie Y. (2015). Designing more sustainable and greener self-compacting concrete. Constr. Build. Mater..

[67] Aman A.M.N., Selvarajoo A., Lau T.L., Chen W.H. (2022). Biochar as cement replacement to enhance concrete composite properties: A review. Energies.

[68] Zhang D., Jaworska B., Zhu H., Dahlquist K., Li V.C. (2020). Engineered Cementitious Composites (ECC) with lime-stone calcined clay cement (LC3). Cem. Concr. Compos..

[69] Shi Y., Long G., Ma C., Xie Y., He J. (2019). Design and preparation of ultra-high performance concrete with low environmental impact. J. Clean. Prod..

[70] Alsalman A., Dang C.N., Martí-Vargas J.R., Hale W.M. (2019). Mixture-proportioning of economical UHPC mixtures. J. Build. Eng..

[71] Adamu M., Mohammed B.S., Liew M.S. (2018). Mechanical properties and performance of high volume fly ash roller compacted concrete containing crumb rubber and nano silica. Constr. Build. Mater..

[72] https://www.dailymetalprice.com/metalpricecharts.php?c=st&u=mt&d=240.

[73] Fantilli A., Mancinelli O., Chiaia B. (2019). The carbon footprint of normal and high-strength concrete used in low-rise and high-rise buildings. Case Stud. Constr. Mater..

