# Supplementary material for: Valorization of Vetiver Root Biochar in Eco-Friendly Reinforced Concrete: Mechanical, Economic, and Environmental Performance
Source: Materials (Basel). 2023 Mar 22;16(6):2522. doi: 10.3390/ma16062522 (PMC10056510; doi:10.3390/ma16062522)
Supplement: Supplementary file 1 [file materials-16-02522-s001.zip › materials-2259702-supplementary.pdf]

materials

MDPI

Supplementary Materials

Table S1. Results of the SPLP test (unit: μg/L).

Age	Metal<br>EPA limit	Hg<br>200	As<br>5000	Se<br>1000	Pb<br>5000	Cd<br>1000	Ag<br>5000	Ba<br>100,000	Mn<br>5000	Cu<br>NA	Zn<br>NA
1 day	Control	BDL	11.09	4.36	BDL	0.40	BDL	31.84	66.64	BDL	BDL
	PBC 2%	BDL	2.51	BDL	BDL	0.76	BDL	26.74	42.52	BDL	BDL
	PBC 4%	BDL	21.73	31.70	BDL	0.58	BDL	40.83	107.5	BDL	BDL
	PBC 6%	BDL	35.94	59.67	BDL	0.34	BDL	49.66	168.3	BDL	BDL
	CBC 2%	3.28	BDL	1.42	BDL	0.62	BDL	49.64	BDL	0.11	BDL
	CBC 4%	BDL	BDL	3.58	BDL	0.82	BDL	28.02	BDL	1.03	BDL
	CBC 6%	BDL	BDL	5.65	BDL	0.97	BDL	14.25	BDL	1.55	BDL
14 days	Control	BDL	BDL	0.09	BDL	1.02	BDL	101.49	868.5	3.08	BDL
	PBC 2%	BDL	BDL	BDL	BDL	0.70	BDL	52.31	281.4	3.66	BDL
	PBC 4%	BDL	BDL	BDL	BDL	1.00	BDL	57.02	349.4	4.96	BDL
	PBC 6%	BDL	BDL	BDL	BDL	1.36	BDL	62.33	503.1	6.87	BDL
	CBC 2%	BDL	BDL	BDL	BDL	0.77	BDL	36.95	149.7	3.36	BDL
	CBC 4%	BDL	BDL	BDL	BDL	0.92	BDL	40.51	199.0	3.28	BDL
	CBC 6%	BDL	BDL	BDL	BDL	1.11	BDL	42.15	248.6	3.02	BDL
28 days	Control	BDL	BDL	BDL	BDL	0.88	BDL	30.60	137.6	BDL	BDL
	PBC 2%	BDL	BDL	BDL	BDL	0.88	BDL	30.38	150.7	BDL	BDL
	PBC 4%	BDL	BDL	BDL	BDL	0.98	BDL	19.98	41.59	BDL	BDL
	PBC 6%	BDL	BDL	BDL	BDL	1.06	BDL	9.55	14.44	BDL	BDL
	CBC 2%	BDL	BDL	BDL	BDL	1.36	BDL	65.71	1141	BDL	BDL
	CBC 4%	BDL	BDL	BDL	BDL	1.39	BDL	42.90	196.7	BDL	BDL
	CBC 6%	BDL	BDL	BDL	BDL	1.46	BDL	34.11	3.22	BDL	BDL

Note: BDL stands for below detection limit.

Table S2. Results of the TCLP test (unit: μg/L).

Age	Metal type<br>EPA limit	Hg<br>200	As<br>5000	Se<br>1000	Pb<br>5000	Cd<br>1000	Ag<br>5000	Ba<br>100,000	Mn<br>5000	Cu<br>NA	Zn<br>NA
1 day	Control	5.73	6.39	5.88	BDL	0.54	BDL	260.90	587	BDL	BDL
	PBC 2%	1.27	2.83	3.54	BDL	0.64	BDL	206.93	573.6	BDL	BDL
	PBC 4%	BDL	7.43	BDL	BDL	0.67	BDL	200.93	611.5	BDL	BDL
	PBC 6%	BDL	8.46	BDL	BDL	0.69	BDL	195.36	735.1	BDL	BDL
	CBC 2%	BDL	13.83	3.07	BDL	0.91	BDL	313.27	1157	BDL	BDL
	CBC 4%	BDL	0.90	6.02	BDL	0.80	BDL	154.01	541.8	BDL	BDL
	CBC 6%	BDL	1.20	9.14	BDL	0.77	BDL	98.47	134.1	BDL	BDL
14 days	Control	BDL	5.28	BDL	BDL	0.59	BDL	32.76	89.91	BDL	BDL
	PBC 2%	BDL	4.93	BDL	BDL	0.68	BDL	23.63	72.04	0.05	BDL
	PBC 4%	BDL	7.82	BDL	BDL	0.75	BDL	27.32	61.4	0.21	BDL
	PBC 6%	BDL	9.64	BDL	BDL	0.88	BDL	32.65	42.31	0.47	BDL
	CBC 2%	BDL	2.35	BDL	BDL	0.65	BDL	19.00	44.89	1.17	BDL
	CBC 4%	BDL	BDL	3.64	BDL	0.66	BDL	22.05	43.6	1.15	BDL
	CBC 6%	BDL	BDL	6.18	BDL	0.60	BDL	25.63	38.34	1.08	BDL
28 days	Control	BDL	0.45	2.04	BDL	1.04	BDL	10.35	24.76	0.24	BDL
	PBC 2%	BDL	12.3	BDL	BDL	1.04	BDL	13.96	31.94	2.18	BDL
	PBC 4%	BDL	5.84	BDL	BDL	1.02	BDL	34.63	204.7	BDL	BDL
	PBC 6%	BDL	6.31	BDL	BDL	1.00	BDL	54.96	198.6	BDL	BDL
	CBC 2%	BDL	BDL	22.57	BDL	0.98	BDL	13.21	38.36	3.65	BDL
	CBC 4%	BDL	2.87	2.15	BDL	0.85	BDL	10.91	39.04	6.1	BDL
	CBC 6%	BDL	BDL	0.15	BDL	0.78	BDL	8.46	41.23	7.84	BDL

Note: BDL stands for below detection limit.

Table S3. Progressive TCLP data at 1 day (unit: μg/L).

	**Run**	**Hg**	**As**	**Ba**	**Se**	**Pb**	**Cd**	**Ag**	**Mn**	**Cu**	**Zn**
Control	1	5.73	6.39	260.9	5.88	BDL	0.54	BDL	587	BDL	BDL
	2	BDL	5.71	355.6	BDL	BDL	0.73	BDL	1741	BDL	BDL
	3	BDL	11.69	494.8	BDL	BDL	1.48	BDL	2151	BDL	BDL
	4	BDL	2.44	508.8	BDL	BDL	1.36	BDL	2000	BDL	BDL
	5	BDL	1.06	562.9	BDL	BDL	1.54	BDL	2011	BDL	BDL
PBC 2%	1	1.27	2.83	206.9	3.54	BDL	0.64	BDL	573.6	BDL	BDL
	2	BDL	BDL	450.3	BDL	BDL	0.98	BDL	1988	BDL	BDL
	3	BDL	BDL	413.7	BDL	BDL	1.01	BDL	1613	BDL	BDL
	4	BDL	BDL	460.9	BDL	BDL	1.24	BDL	1696	BDL	BDL
	5	BDL	BDL	497.6	BDL	BDL	1.83	BDL	1738	BDL	BDL
PBC 4%	1	BDL	7.43	200.9	BDL	BDL	0.67	BDL	565	BDL	BDL
	2	BDL	3.06	119.3	0.27	BDL	0.71	BDL	611.6	BDL	BDL
	3	BDL	BDL	256.8	BDL	BDL	0.88	BDL	1538	BDL	BDL
	4	BDL	BDL	422.5	BDL	BDL	1.16	BDL	1778	BDL	BDL
	5	BDL	BDL	438.2	BDL	BDL	1.43	BDL	1828	BDL	BDL
PBC 6%	1	BDL	13.25	196.9	BDL	BDL	0.737	BDL	553.7	BDL	BDL
	2	BDL	5.66	116.9	BDL	BDL	0.781	BDL	599.3	BDL	BDL
	3	BDL	2.1	251.7	BDL	BDL	0.968	BDL	1507	BDL	BDL
	4	BDL	BDL	414.1	BDL	BDL	1.276	BDL	1742	BDL	BDL
	5	BDL	BDL	429.5	BDL	BDL	1.573	BDL	1791	BDL	BDL
CBC 2%	1	BDL	BDL	313.3	BDL	BDL	0.91	BDL	1157	BDL	BDL
	2	BDL	BDL	119	BDL	BDL	0.91	BDL	589.4	BDL	BDL
	3	BDL	5.51	416.6	BDL	BDL	0.95	BDL	1630	BDL	BDL
	4	BDL	3.84	525.9	BDL	BDL	1.35	BDL	1976	BDL	BDL
	5	BDL	2.83	613.3	BDL	BDL	1.67	BDL	2031	BDL	BDL

CBC 4%	1	BDL	0.9	154	6.02	BDL	0.8	BDL	541.9	BDL	BDL
	2	BDL	5.9	202.7	BDL	BDL	0.86	BDL	1013	BDL	BDL
	3	BDL	12.41	385.4	BDL	BDL	1.1	BDL	1508	BDL	BDL
	4	BDL	20.46	442.9	BDL	BDL	1.21	BDL	1566	BDL	BDL
	5	BDL	28.64	503.2	BDL	BDL	1.56	BDL	1624	BDL	BDL
CBC 6%	1	BDL	6.3	92.41	BDL	BDL	0.72	BDL	243.9	BDL	BDL
	2	BDL	41.3	121.6	BDL	BDL	0.774	BDL	455.9	BDL	BDL
	3	BDL	86.87	231.3	BDL	BDL	0.99	BDL	678.8	BDL	BDL
	4	BDL	143.2	265.8	BDL	BDL	1.089	BDL	704.7	BDL	BDL
	5	BDL	200.5	301.9	BDL	BDL	1.404	BDL	731.2	BDL	BDL

Note: BDL stands for below detection limit.

Table S4. Progressive TCLP data at 14 days (unit: μg/L).

	Run	Hg	As	Se	Pb	Cd	Mn	Ba	Ag	Cu	Zn
Control	1	BDL	5.28	BDL	BDL	0.59	89.91	32.76	BDL	BDL	BDL
	2	BDL	5.755	BDL	BDL	0.643	98.01	35.71	BDL	BDL	BDL
	3	BDL	6.273	BDL	BDL	0.701	106.8	38.92	BDL	BDL	BDL
	4	BDL	6.838	BDL	BDL	0.764	116.4	42.42	BDL	BDL	BDL
	5	BDL	7.453	BDL	BDL	0.833	126.9	46.24	BDL	BDL	BDL
PBC 2%	1	BDL	4.93	BDL	BDL	0.68	72.04	23.63	BDL	0.05	BDL
	2	BDL	5.373	BDL	BDL	0.741	78.52	25.75	BDL	BDL	BDL
	3	BDL	5.857	BDL	BDL	0.808	85.59	28.07	BDL	BDL	BDL
	4	BDL	6.385	BDL	BDL	0.881	93.29	30.60	BDL	BDL	BDL
	5	BDL	6.959	BDL	BDL	0.959	101.7	33.36	BDL	BDL	BDL
PBC 4%	1	BDL	7.82	BDL	BDL	0.75	61.4	27.32	BDL	0.21	BDL
	2	BDL	8.524	BDL	BDL	0.817	66.92	29.78	BDL	BDL	BDL
	3	BDL	9.291	BDL	BDL	0.891	72.95	32.46	BDL	BDL	BDL
	4	BDL	10.13	BDL	BDL	0.971	79.52	35.38	BDL	BDL	BDL
	5	BDL	11.04	BDL	BDL	1.059	86.67	38.56	BDL	BDL	BDL
PBC 6%	1	BDL	10.17	BDL	BDL	0.975	50.35	30.05	BDL	0.61	BDL
	2	BDL	11.08	BDL	BDL	1.062	54.88	32.76	BDL	BDL	BDL
	3	BDL	12.07	BDL	BDL	1.158	59.82	35.71	BDL	BDL	BDL
	4	BDL	13.16	BDL	BDL	1.262	65.20	38.91	BDL	BDL	BDL
	5	BDL	14.35	BDL	BDL	1.376	71.07	42.42	BDL	BDL	BDL
CBC 2%	1	BDL	2.35	BDL	BDL	0.65	44.89	19	BDL	1.17	BDL
	2	BDL	2.562	BDL	BDL	0.708	48.93	20.71	BDL	BDL	BDL
	3	BDL	2.792	BDL	BDL	0.772	53.33	22.57	BDL	BDL	BDL
	4	BDL	3.043	BDL	BDL	0.842	58.13	24.60	BDL	BDL	BDL
	5	BDL	3.317	BDL	BDL	0.917	63.36	26.82	BDL	BDL	BDL
CBC 4%	1	BDL	BDL	3.64	BDL	0.66	43.6	22.05	BDL	1.15	BDL
	2	BDL	BDL	3.967	BDL	BDL	47.52	24.03	BDL	1.253	BDL
	3	BDL	BDL	4.325	BDL	BDL	51.81	26.19	BDL	1.366	BDL
	4	BDL	BDL	4.714	BDL	BDL	56.46	28.55	BDL	1.489	BDL
	5	BDL	BDL	5.138	BDL	BDL	61.54	31.12	BDL	1.623	BDL
CBC 6%	1	BDL	BDL	BDL	BDL	0.68	41.42	22.93	BDL	1.207	BDL
	2	BDL	BDL	BDL	BDL	BDL	45.14	24.99	BDL	1.316	BDL
	3	BDL	BDL	BDL	BDL	BDL	49.21	27.24	BDL	1.434	BDL
	4	BDL	BDL	BDL	BDL	BDL	53.64	29.69	BDL	1.563	BDL
	5	BDL	BDL	BDL	BDL	BDL	58.46	32.37	BDL	1.704	BDL

Note: BDL stands for below detection limit.

Table S5. Progressive TCLP data at 28 days (unit: μg/L).

	Run	Hg	As	Se	Pb	Cd	Mn	Ba	Ag	Cu	Zn

	1	BDL	0.45	2.04	BDL	1.04	24.76	10.35	BDL	0.24	BDL
	2	BDL	0.490	2.22	BDL	1.133	26.98	11.28	BDL	0.261	BDL
Control	3	BDL	0.534	2.42	BDL	1.235	29.41	12.29	BDL	0.285	BDL
	4	BDL	0.582	2.64	BDL	1.346	32.06	13.41	BDL	0.311	BDL
	5	BDL	0.635	2.88	BDL	1.468	34.95	14.61	BDL	0.338	BDL
	1	BDL	12.3	BDL	BDL	1.04	31.94	13.96	BDL	2.18	BDL
	2	BDL	13.41	BDL	BDL	1.133	34.81	15.21	BDL	2.376	BDL
PBC 2%	3	BDL	14.61	BDL	BDL	1.235	37.94	16.58	BDL	2.591	BDL
	4	BDL	15.93	BDL	BDL	1.346	41.36	18.07	BDL	2.823	BDL
	5	BDL	17.36	BDL	BDL	1.468	45.08	19.71	BDL	3.077	BDL
	1	BDL	5.84	BDL	BDL	1.02	204.8	34.63	BDL	BDL	BDL
	2	BDL	6.365	BDL	BDL	1.111	223.2	37.74	BDL	BDL	BDL
PBC 4%	3	BDL	6.938	BDL	BDL	1.211	243.3	41.14	BDL	BDL	BDL
	4	BDL	7.562	BDL	BDL	1.320	265.2	44.84	BDL	BDL	BDL
	5	BDL	8.243	BDL	BDL	1.439	289.1	48.88	BDL	BDL	BDL
	1	BDL	3.971	BDL	BDL	0.969	233.5	69.26	BDL	BDL	BDL
	2	BDL	4.328	BDL	BDL	1.056	254.5	75.49	BDL	BDL	BDL
PBC 6%	3	BDL	4.718	BDL	BDL	1.151	277.4	82.28	BDL	BDL	BDL
	4	BDL	5.142	BDL	BDL	1.254	302.4	89.69	BDL	BDL	BDL
	5	BDL	5.605	BDL	BDL	1.367	329.5	97.76	BDL	BDL	BDL
	1	BDL	BDL	22.57	BDL	0.98	38.36	13.21	BDL	3.65	BDL
	2	BDL	BDL	24.60	BDL	BDL	41.81	14.39	BDL	3.978	BDL
CBC 2%	3	BDL	BDL	26.81	BDL	BDL	45.57	15.69	BDL	4.336	BDL
	4	BDL	BDL	29.22	BDL	BDL	49.67	17.11	BDL	4.726	BDL
	5	BDL	BDL	31.85	BDL	BDL	54.14	18.64	BDL	5.152	BDL
	1	BDL	2.87	2.15	BDL	0.85	39.04	10.91	BDL	6.1	BDL
	2	BDL	3.128	2.343	BDL	BDL	42.55	11.89	BDL	6.649	BDL
CBC 4%	3	BDL	3.409	2.554	BDL	BDL	46.38	12.96	BDL	7.247	BDL
	4	BDL	3.716	2.784	BDL	BDL	50.55	14.12	BDL	7.899	BDL
	5	BDL	4.051	3.034	BDL	BDL	55.11	15.40	BDL	8.611	BDL
	1	BDL	8.61	0.239	BDL	0.64	40.21	9.611	BDL	10.79	BDL
	2	BDL	9.384	0.260	BDL	BDL	43.83	10.46	BDL	11.76	BDL
CBC 6%	3	BDL	10.22	0.284	BDL	BDL	47.77	11.41	BDL	12.82	BDL
	4	BDL	11.15	0.309	BDL	BDL	52.07	12.43	BDL	13.98	BDL
	5	BDL	12.15	0.337	BDL	BDL	56.76	13.55	BDL	15.24	BDL

Note: BDL stands for below detection limit.
